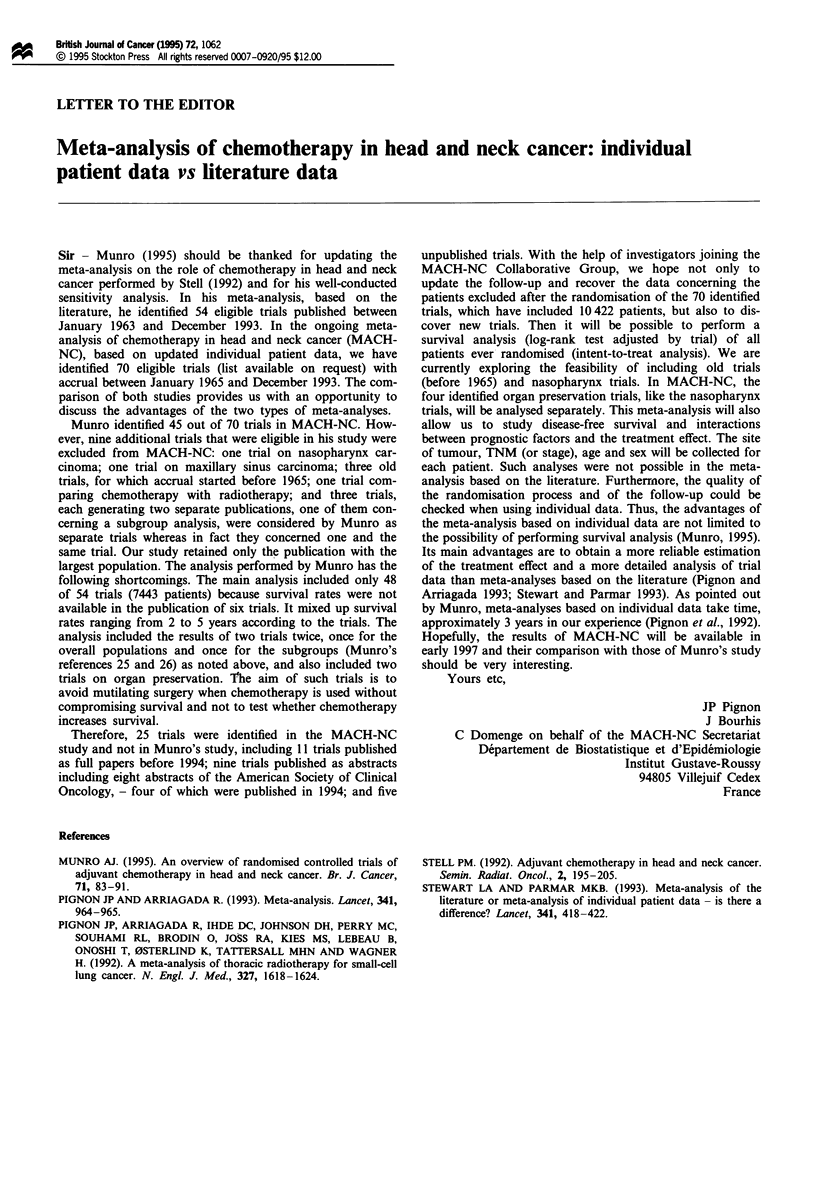# Meta-analysis of chemotherapy in head and neck cancer: individual patient data vs literature data.

**DOI:** 10.1038/bjc.1995.462

**Published:** 1995-10

**Authors:** J. P. Pignon, J. Bourhis


British Journal of Cancer (1995) 72, 1062
© 1995 Stockton Press All rights reserved 0007-0920/95 $12.00

LETTER TO THE EDITOR

### Meta-analysis of chemotherapy in head and neck cancer: individual patient data *vs* literature data

**Sir** – Munro (1995) should be thanked for updating the meta-analysis on the role of chemotherapy in head and neck cancer performed by Stell (1992) and for his well-conducted sensitivity analysis. In his meta-analysis, based on the literature, he identified 54 eligible trials published between January 1963 and December 1993. In the ongoing meta-analysis of chemotherapy in head and neck cancer (MACH-NC), based on updated individual patient data, we have identified 70 eligible trials (list available on request) with accrual between January 1965 and December 1993. The comparison of both studies provides us with an opportunity to discuss the advantages of the two types of meta-analyses.

Munro identified 45 out of 70 trials in MACH-NC. However, nine additional trials that were eligible in his study were excluded from MACH-NC: one trial on nasopharynx carcinoma; one trial on maxillary sinus carcinoma; three old trials, for which accrual started before 1965; one trial comparing chemotherapy with radiotherapy; and three trials, each generating two separate publications, one of them concerning a subgroup analysis, were considered by Munro as separate trials whereas in fact they concerned one and the same trial. Our study retained only the publication with the largest population. The analysis performed by Munro has the following shortcomings. The main analysis included only 48 of 54 trials (7443 patients) because survival rates were not available in the publication of six trials. It mixed up survival rates ranging from 2 to 5 years according to the trials. The analysis included the results of two trials twice, once for the overall populations and once for the subgroups (Munro's references 25 and 26) as noted above, and also included two trials on organ preservation. The aim of such trials is to avoid mutilating surgery when chemotherapy is used without compromising survival and not to test whether chemotherapy increases survival.

Therefore, 25 trials were identified in the MACH-NC study and not in Munro's study, including 11 trials published as full papers before 1994; nine trials published as abstracts including eight abstracts of the American Society of Clinical Oncology, – four of which were published in 1994; and five unpublished trials. With the help of investigators joining the MACH-NC Collaborative Group, we hope not only to update the follow-up and recover the data concerning the patients excluded after the randomisation of the 70 identified trials, which have included 10 422 patients, but also to discover new trials. Then it will be possible to perform a survival analysis (log-rank test adjusted by trial) of all patients ever randomised (intent-to-treat analysis). We are currently exploring the feasibility of including old trials (before 1965) and nasopharynx trials. In MACH-NC, the four identified organ preservation trials, like the nasopharynx trials, will be analysed separately. This meta-analysis will also allow us to study disease-free survival and interactions between prognostic factors and the treatment effect. The site of tumour, TNM (or stage), age and sex will be collected for each patient. Such analyses were not possible in the meta-analysis based on the literature. Furthermore, the quality of the randomisation process and of the follow-up could be checked when using individual data. Thus, the advantages of the meta-analysis based on individual data are not limited to the possibility of performing survival analysis (Munro, 1995). Its main advantages are to obtain a more reliable estimation of the treatment effect and a more detailed analysis of trial data than meta-analyses based on the literature (Pignon and Arriagada 1993; Stewart and Parmar 1993). As pointed out by Munro, meta-analyses based on individual data take time, approximately 3 years in our experience (Pignon *et al.*, 1992). Hopefully, the results of MACH-NC will be available in early 1997 and their comparison with those of Munro's study should be very interesting.

Yours etc,

JP Pignon
J Bourhis
C Domenge on behalf of the MACH-NC Secretariat
Département de Biostatistique et d'Epidémiologie
Institut Gustave-Roussy
94805 Villejuif Cedex
France

**References**

MUNRO AJ. (1995). An overview of randomised controlled trials of adjuvant chemotherapy in head and neck cancer. *Br. J. Cancer*, **71**, 83–91.

PIGNON JP AND ARRIAGADA R. (1993). Meta-analysis. *Lancet*, **341**, 964–965.

PIGNON JP, ARRIAGADA R, IHDE DC, JOHNSON DH, PERRY MC, SOUHAMI RL, BRODIN O, JOSS RA, KIES MS, LEBEAU B, ONOSHI T, ØSTERLIND K, TATTERSALL MHN AND WAGNER H. (1992). A meta-analysis of thoracic radiotherapy for small-cell lung cancer. *N. Engl. J. Med.*, **327**, 1618–1624.

STELL PM. (1992). Adjuvant chemotherapy in head and neck cancer. *Semin. Radiat. Oncol.*, **2**, 195–205.

STEWART LA AND PARMAR MKB. (1993). Meta-analysis of the literature or meta-analysis of individual patient data – is there a difference? *Lancet*, **341**, 418–422.